# Development of Newly Synthesized Chromone Derivatives with High Tumor Specificity against Human Oral Squamous Cell Carcinoma

**DOI:** 10.3390/medicines7090050

**Published:** 2020-08-26

**Authors:** Yoshiaki Sugita, Koichi Takao, Yoshihiro Uesawa, Junko Nagai, Yosuke Iijima, Motohiko Sano, Hiroshi Sakagami

**Affiliations:** 1 Department of Pharmaceutical Sciences, Faculty of Pharmacy and Pharmaceutical Sciences, Josai University, Saitama 350-0295, Japan; ktakao@josai.ac.jp; 2 Department of Medical Molecular Informatics, Meiji Pharmaceutical University, Tokyo 204-858, Japan; nagai-j@my-pharm.ac.jp; 3 Department of Oral and Maxillofacial Surgery, Saitama Medical Center, Saitama Medical University, Kawagoe 350-8550, Japan; yoiijima@saitama-med.ac.jp; 4 Division of Applied Pharmaceutical Education and Research, Hoshi University, Tokyo 142-8501, Japan; m-sano@hoshi.ac.jp; 5 Meikai University Research Institute of Odontology (M-RIO), 1-1 Keyakidai, Sakado, Saitama 350-0283, Japan

**Keywords:** chromone, tumor specificity, QSAR analysis, apoptosis, cell cycle analysis

## Abstract

Since many anticancer drugs show severe adverse effects such as mucositis, peripheral neurotoxicity, and extravasation, it was crucial to explore new compounds with much reduced adverse effects. Comprehensive investigation with human malignant and nonmalignant cells demonstrated that derivatives of chromone, back-bone structure of flavonoid, showed much higher tumor specificity as compared with three major polyphenols in the natural kingdom, such as lignin-carbohydrate complex, tannin, and flavonoid. A total 291 newly synthesized compounds of 17 groups (consisting of 12 chromones, 2 esters, and 3 amides) gave a wide range of the intensity of tumor specificity, possibly reflecting the fitness for the optimal 3D structure and electric state. Among them, 7-methoxy-3-[(1*E*)-2-phenylethenyl]-4*H*-1-benzopyran-4-one (compound **22**), which belongs to 3-styrylchromones, showed the highest tumor specificity. **22** induced subG1 and G2 + M cell population in human oral squamous cell carcinoma cell line, with much less keratinocyte toxicity as compared with doxorubicin and 5-FU. However, 12 active compounds selected did not necessarily induce apoptosis and mitotic arrest. This compound can be used as a lead compound to manufacture more active compound.

## 1. Introduction

This review is composed of four parts. The first part reviews the adverse effect of chemotherapeutic agents. The second part introduces our life-work research of development of chromone derivatives that show comparable anticancer activity and lower keratinocyte toxicity, as compared with anticancer drug. The third part describes the serious problems of neurotoxicity in G2 + M blocker. The fourth part is the summary of our major findings and future direction of chromone research.

## 2. Adverse Effects of Anticancer Drugs

### 2.1. Oral Mucositis Associated with Anticancer Drug

Oral mucositis is one of the most frequent adverse events in cancer drug therapy and hematopoietic stem cell transplantation. Oral mucositis is reported to occur in 5–50% of patients receiving standard-dose chemotherapy and 68–98% of high-dose chemotherapy related to hematopoietic stem cell transplantation [1]. Oral mucositis not only lowers the patient’s QOL due to pain but also lowers the oral intake, leads to undernutrition and dehydration, and deteriorates the general condition. It also serves as a gateway for bacterial invasion and may trigger systemic infection. Decreased doses and delayed schedules in chemotherapy lead to reduced efficacy and survival rates, but currently there are no established preventive or curative methods for oral mucositis, hindering smooth cancer treatment [2].

### 2.2. Neurotoxicity of Anticancer Drugs

Cancer drug therapy has contributed to the improvement of survival rate and QOL by the development of cytocidal anticancer drugs and molecular targeted therapeutic agents, while the adverse effect of cancer drug therapy causes a decrease in QOL, sometimes causing the discontinuation of the drug therapy. Typical side effects include organ disorders such as bone marrow suppression, physical disorders such as nausea and vomiting, and neuropathy represented by paresthesia. Chemotherapy-induced peripheral neuropathy (CIPN) associated with an anticancer agent is not recovered quickly by a drug withdrawal like myelosuppression, and some disorders may remain for the lifetime. CIPN is a serious adverse event that interferes with the continuation of chemotherapy. It was reported that the incidence of CIPN was 68.1% within 1 month after chemotherapy, 60.0% after 3 months, and 30.0% after 6 months in a follow-up study of 4179 patients with colorectal cancer, breast cancer, gynecologic cancer, and multiple myeloma [3]. However, there are a few reports of preventive and therapeutic drugs for CIPN. Platinum, taxane, and vinca alkaloid are known as causative agents of CIPN. Different drugs have different mechanisms that cause peripheral neuropathy. For example, platinum-based cisplatin causes sensorineural deafness in the high range due to acoustic nerve damage. It has been reported that it is cumulative and that symptoms often continue for a long period of time after discontinuation of administration [4].

Oxaliplatin, a platinum drug, has acute and chronic symptoms. Acute symptoms are characterized by paresthesia around the extremities and around the lips and chronic symptoms may persist for months to years [5]. Carboplatin, a platinum drug, causes relatively few neurological symptoms when used at normal doses, and high doses may cause symptoms similar to cisplatin [6]. Paclitaxel, a taxane-based drug, mainly causes paresthesia of the extremities and is correlated with single dose and total dose [7]. Docetaxel, a taxane-based drug, causes sensory and motor disorders but is less frequent than paclitaxel [8]. Vincristine, a vinca alkaloid drug, causes sensory abnormalities in the fingers and movements within a few weeks after the start of treatment and often persists for a long time after the discontinuation of treatment [9]. CIPN pathological findings are classified into axonopathy, neuronopathy, and myelinopathy. Axonopathy is the most common disorder in CIPN. Neuronal cell body is relatively retained due to damage from thick and long axons. Clinically, glove and stocking type sensory deficits often begin at the extremities. Representative agents are microtubule inhibitors, vinca alkaloids, and taxanes. Neuronopathy is mainly cell bodies of lesions, mainly caused by cell death of dorsal root ganglion cells, and secondary damage to axons and myelin sheaths. Clinically, nerve cell bodies with short axons are also damaged, so sensory deficits occur not only on the extremities but also on the trunk and face. Representative agents are platinum agents such as oxaliplatin and cisplatin [10]. The frequency of mucositis and peripheral neuropathy of various anticancer agents was summarized from the interview form of the pharmaceutical company (Table 1).

### 2.3. Chemotherapy Extravasation

Systemic intravenous chemotherapy can cause multiple emergencies by local and systemic reactions. Drug extravasation is one of the most devastating complications in chemotherapy. Overall incidences of chemotherapy extravasation ranges from 0.01% to 6.5% [11,12,13], with reports of extravasation occurrence via central venous catheters ranging from 0.3% to 10.3% [14,15,16,17]. The exact incidence rate of extravasation varies greatly due to the general lack of reporting and absence of centralized registry of extravasation events. Therefore, no benchmark existed for the incidence of chemotherapy extravasations.

Extravasation is the accidental leakage of cytotoxic chemotherapy drugs that can cause severe tissue damage, tissue necrosis, blistering, or sloughing into the subcutaneous or subdermal tissue at the injection site [18,19]. Extravasated drugs are further classified into the three groups: vesicants, irritants, and nonvesicants/nonirritants, according to their potential for causing damage as (Table 2) [12,13,19,20]. 

Vesicant drugs have the capability to induce the formation of blisters and/or cause tissue destruction. Vesicant drugs may be subclassified into DNA-binding and non-DNA-binding compounds [20]. DNA-binding compounds are capable of producing more severe tissue damage and mainly include anthracyclines and alkylating agents. Non-DNA-binding compounds are mainly vinca alkaloids and taxanes. 

Irritant drugs can cause pain at the injection site or along the vein, with or without an inflammatory reaction. Some of these agents have the potential to cause soft tissue ulcers only if a large amount of concentrated drug solution is inadvertently extravasated. Nonvesicant or nonirritant drugs, if extravasated, rarely produce acute reactions or tissue necrosis. 

Tissue damage related to extravasation occurs by different mechanisms [3]. First, the drug is absorbed by local cells in the tissue and binds to critical structures, causing cell death. After the endocytolysis, surrounding cells can also die through the release of the drug from nearby dead cells. The repetitive nature of this process impairs healing and may result in progressive and chronic tissue injury. Second, the drug that does not bind to cellular DNA may metabolize and be cleared, limiting the degree of tissue injury [21]. However, the literature addressing extravasation is limited to animal studies, case reports, and small human studies. Classic randomized studies in humans for the treatment of extravasations are unthinkable because of ethical reasons. On the whole, the highest possible grade of recommendation of each measure for extravasations would be low. Novel studies are clearly needed to elucidate the mechanism of chemotherapy extravasations.

## 3. Development of Newly Synthesized Chromone Derivatives with High Tumor Specificity, but Low Keratinocyte Toxicity

**Figure 1:**
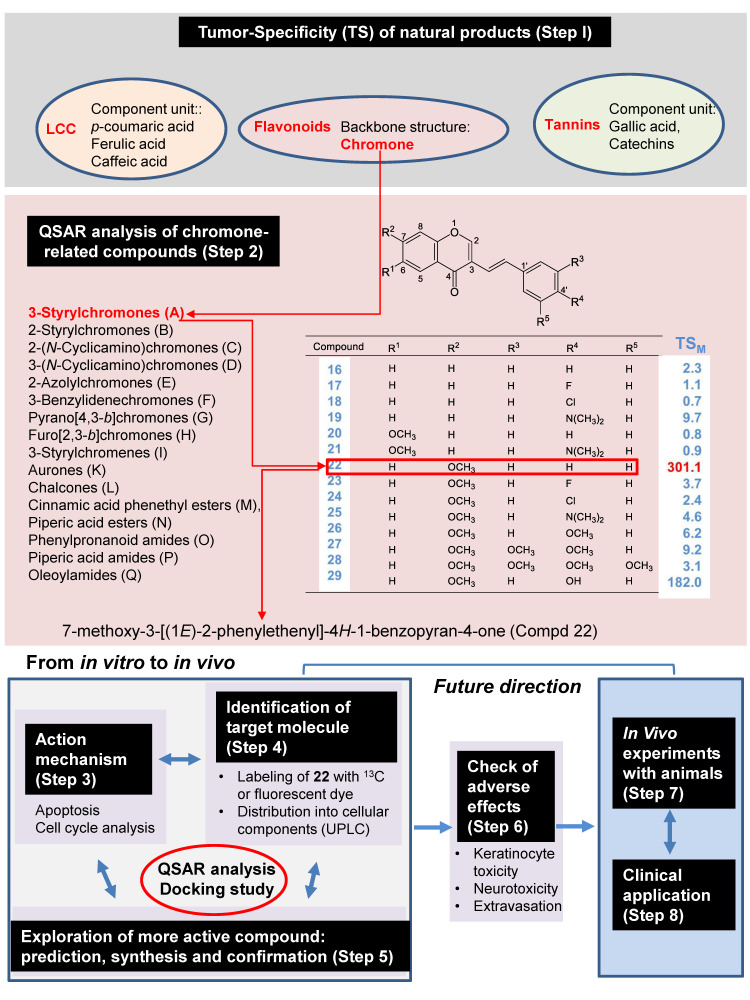
Strategy for exploring new anticancer drugs using chromone backbone structure.

Our strategy to explore new compounds is composed with the following eight steps: search of natural products that shows the highest tumor specificity (Step 1); QSAR analysis of chromone-related compounds (Step 2); investigation of action mechanism (Step 3), identification of target molecules (Step 4), exploration of more activity compounds by prediction, synthesis, and confirmation (Step 5); check for adverse effects (Step 6); in vivo experiments with animals (Step 7); and clinical application (Step 8) (Figure 1).

### 3.1. Why We Focused on the Chromones

**Figure 2:**
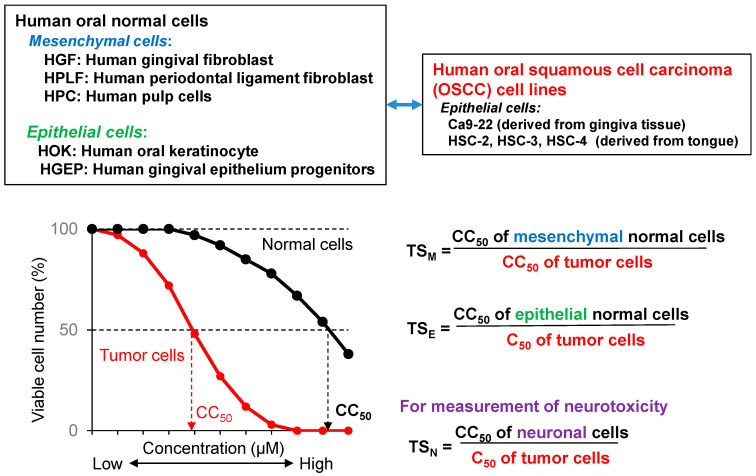
In vitro assay system for the measurement of tumor specificity.

For the quantification of anticancer activity of text samples, it was necessary to establish the in vitro assay method (Figure 2) [22], using human malignant and nonmalignant cells: four human oral squamous cell carcinoma (OSCC) cell lines (Ca9-22, HSC-2, HSC-3, and HSC-4), three human normal oral mesenchymal cells (gingival fibroblast HGF, periodontal ligament fibroblast HPLF, and pulp cell HPC), and two human normal oral epithelial cells (human oral keratinocyte HOK and primary human gingival epithelial cells HGEP). 

**Figure 3:**
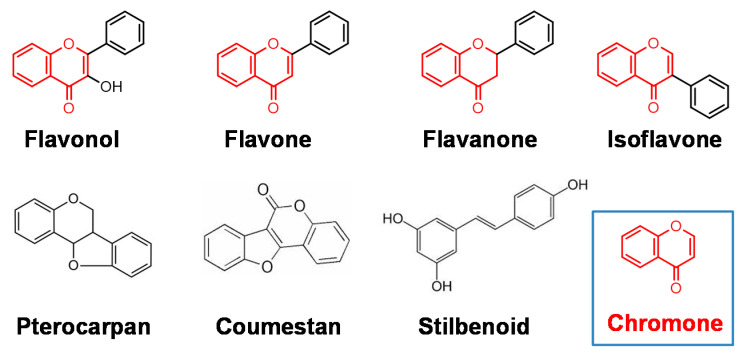
Chromone is a backbone structure of some flavonoids.

Tumor specificity (TS) was defined as the ratio of the mean of CC_50_ against normal cells to that against OSCC cells). When mesenchymal or epithelial cells were used, TS_M_ and TS_E_ could be obtained, respectively. TS_E_ can be used as an index for neurotoxicity. (Figure 1). It would be the most ideal if we could use human epithelial cells as target cells. However, most of anticancer drugs show potent cytotoxicity against epithelial cells (as described later). Therefore, we used TS_M_ value, rather than TS_E_ at the first stage of random screening. Using this method, we found that three major polyphenols, i.e., lignin–carbohydrate complexes, tannins, and flavonoids, showed much lower TS_M_ in comparison to popular chemotherapeutic antitumor drugs (Table 3). On the other hand, the derivatives of chromone, the backbone structure of various flavonoids such as flavonoid, flavone, flavanone, and isoflavone (Figure 3) showed much higher TS_M_ than the majority of polyphenols [22]. These findings encouraged us to explore more active chromone derivatives. 

### 3.2. Synthesis of Chromones, Esters, and Amides

**Figure 4:**
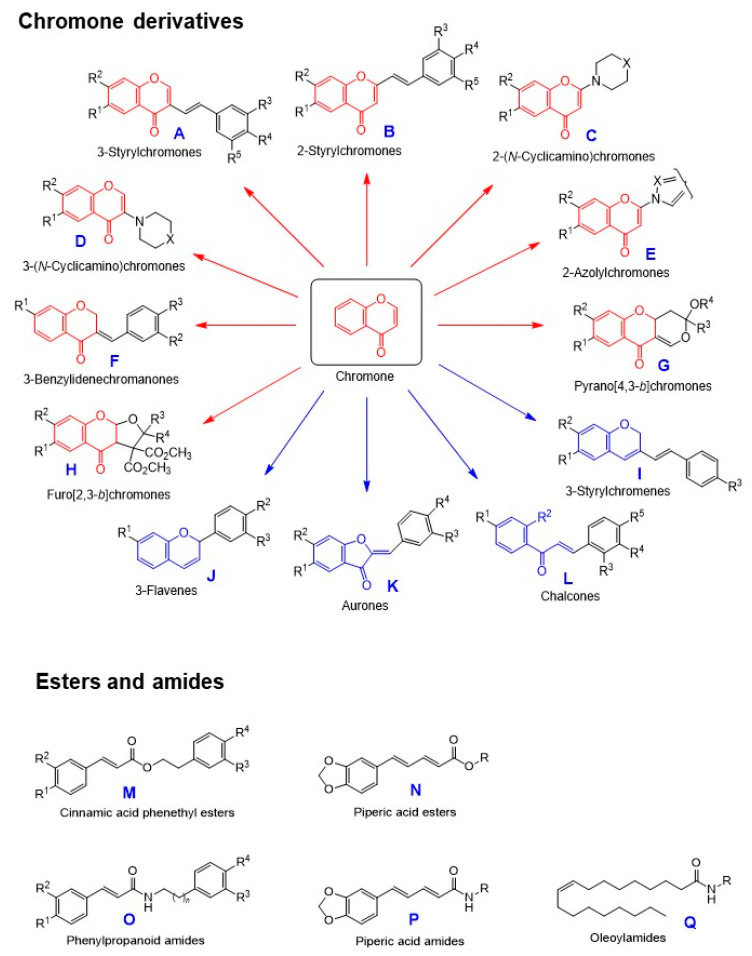
Structure of chromones (**A**~**L**), esters (**M**,**N**), and amides (**O**~**Q**).

We have focused on the following three groups of compounds (Figure 4):

- **Chromone derivatives:**
  **having intact chromone ring:** 3-styrylchromones (**A**), 2-styrylchromones (**B**), 2-(*N*-cyclicamino)chromones (**C**), 3-(*N*-cyclicamino)chromones (**D**), 2-azolylchromones (**E**), 3-benzylidenechromones (**F**), pyrano[4,3-*b*]chromones (**G**), furo[2,3-*b*]chromones (**H**).
  **having chromen ring:** 3-styrylchromenes (**I**) and 3-flavens (**J**) (unpublished).
  **having cleaved chromone ring:** aurones (**K**) and chalcones (**L**).
- **Esters**: cinnamic acid phenethyl esters (**M**) and piperic acid esters (**N**).
- **Amides:** phenylpronanoid amides (**O**), piperic acid amides (**P**), and oleoylamides (**Q**) (Figure 4).

**Figure 5:**
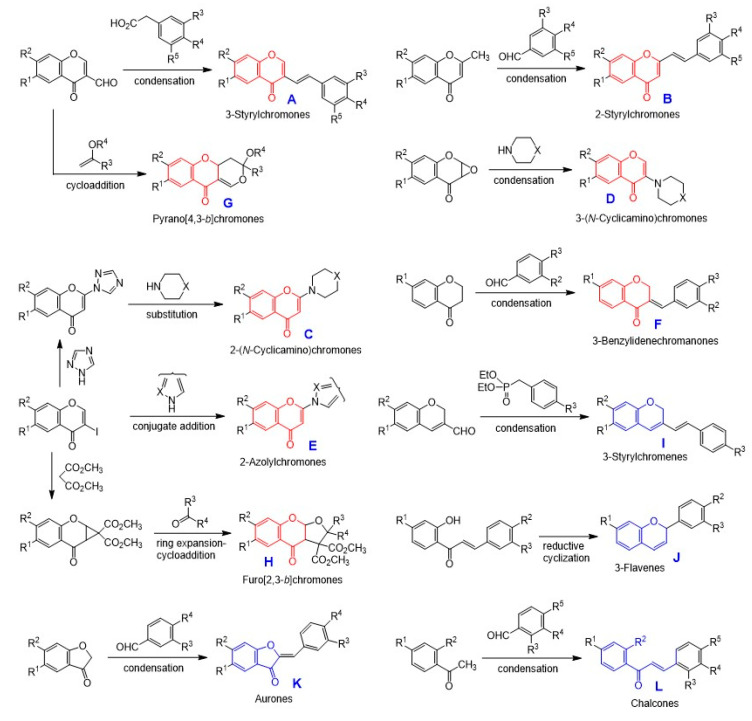
Synthesis of chromone derivatives.

As for chromone derivatives, 3-styrylchromones (**A**) were synthesized by Knoevenagel condensation of the corresponding 3-formylchromones with various phenylacetic acid derivatives [23] (Figure 5).

Here, 2-styrylchromones (**B**) were synthesized by base-catalyzed condensation of the corresponding 2-methylchromones with selected benzaldehyde derivatives [24].

Then, 2-(*N*-cyclicamino)chromones (**C**) were synthesized by the nucleophilic substitution reaction of 2-triazolylchromone derivatives, derived from 3-iodochromones and triazole, with the cyclic secondary amines such as piperidine and piperazine derivatives [25].

Then, 3-(*N*-cyclicamino)chromones (**D**) were synthesized by the condensation of 2,3-epoxychromone derivatives with the cyclic secondary amines [25].

Then, 2-azolylchromones (**E**) were synthesized by the conjugated addition reaction of 3-iodochromone derivatives with various azoles [26].

Next, 3-benzylidenechromones (**F**) were synthesized by base-catalyzed condensation of the corresponding 4-chromanone with substituted benzaldehyde derivatives [27].

Pyrano[4,3*-b*]chromones (**G**) were synthesized by the cycloaddition reaction of 3-formylchromones with selected enol ethers [28].

Furo[2,3-*b*]chromones (**H**) were synthesized by the ring expansion-cycloaddition reaction of methanochromanones with aldehydes or ketones [29].

Basically, 3-styrylchromenes (**I**) were synthesized by Horner-Wadsworth-Emmons reaction of the corresponding 2*H*-chromene-3-carbaldehydes with commercially available diethyl benzylphosphonate derivatives [30]. Additionally, 3-flavens (**J**) were synthesized by reductive intramolecular cycloaddition reaction of 2-hydroxychalcone derivatives [31]. Aurones (**K**) were synthesized by base-catalyzed condensation of 3(2*H*)-benzofuranones with selected benzaldehyde derivatives [32]. Chalcones (**L**) were synthesized by base-catalyzed condensation of the corresponding acetophenones with various benzaldehyde derivatives [31] (Figure 5).

As for esters and amides, cinnamic acid phenethyl esters (**M**) were synthesized by the condensation of cinnamic acid and its analogs, such as caffeic acid, ferulic acid, and *p*-coumaric acid, with the corresponding phenethyl alcohols. In addition, phenylpropanoid amides (**O**) were synthesized by the condensation of the corresponding cinnamic acid derivatives with various biogenic amines.

Piperic acid esters (**N**) were synthesized by the condensation of piperic acid with the corresponding alcohols. In addition, piperic acid amides (**P**) were synthesized by the condensation of the acid chloride of piperic acid with various amines. Piperic acid was prepared by alkaline hydrolysis of piperine.

**Figure 6:**
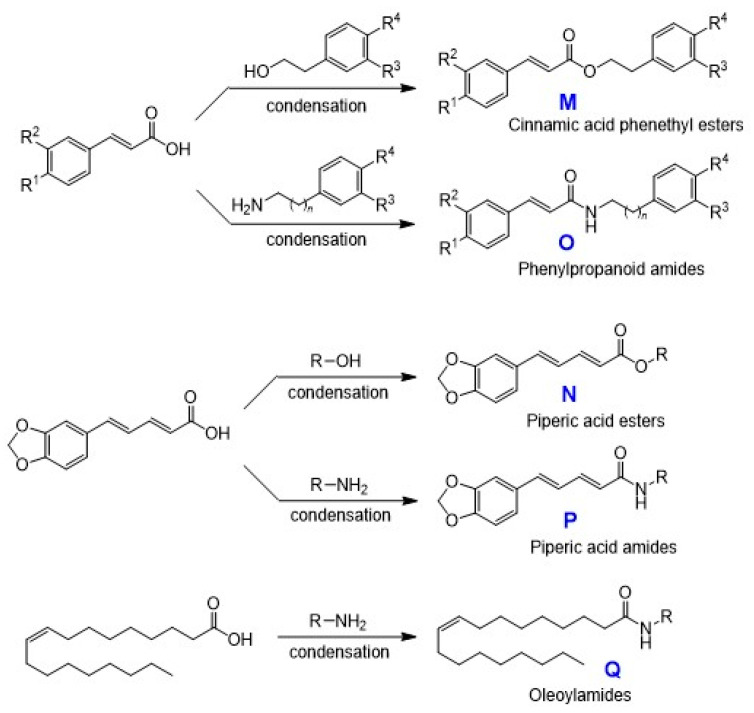
Synthesis of esters and amides.

Oleoylamides (**Q**) were synthesized by the condensation of oleoyl chloride, derived from oleic acid and oxalyl chloride, with the various corresponding biogenic amines (Figure 6).

### 3.3. Tumor Specificity of Chromones, Esters, and Amides

We investigated a total 291 compounds from 17 different groups (**A~Q**) of their cytotoxicity (assessed by CC_50_) against four human OSCC (Ca9-22, HSC-2, HSC-3, and HSC-4) and three human normal mesenchymal cells (HGF, HPLF, and HPC), and then their tumor specificity (assessed by TS_M_, calculated as describe in Figure 2, and potency-selectivity expression (PSE)) [33,34,35,36,37,38,39,40,41,42,43,44,45,46,47,48,49,50,51,52]. PSE, that reflects both tumor specificity and cytotoxicity against tumor cells, was calculated by dividing the TS_M_ by CC_50_ for tumor cells, and then multiplying by 100. All these values are listed in Supplementary Table S1. This demonstrated that only limited numbers of compounds show higher tumor specificity, although their structures are very similar with each other. It is possible that such highly tumor-specific compounds show the optimal 3D structure, since the tumor specificity of chromone compounds shows the tight correlation with chemical descriptors that reflect the 3-D structure (Table 4) [33,35,37,38,39,40,41,42,43,44,45,46,47,48,49,50,51,52,53].

**Figure 7:**
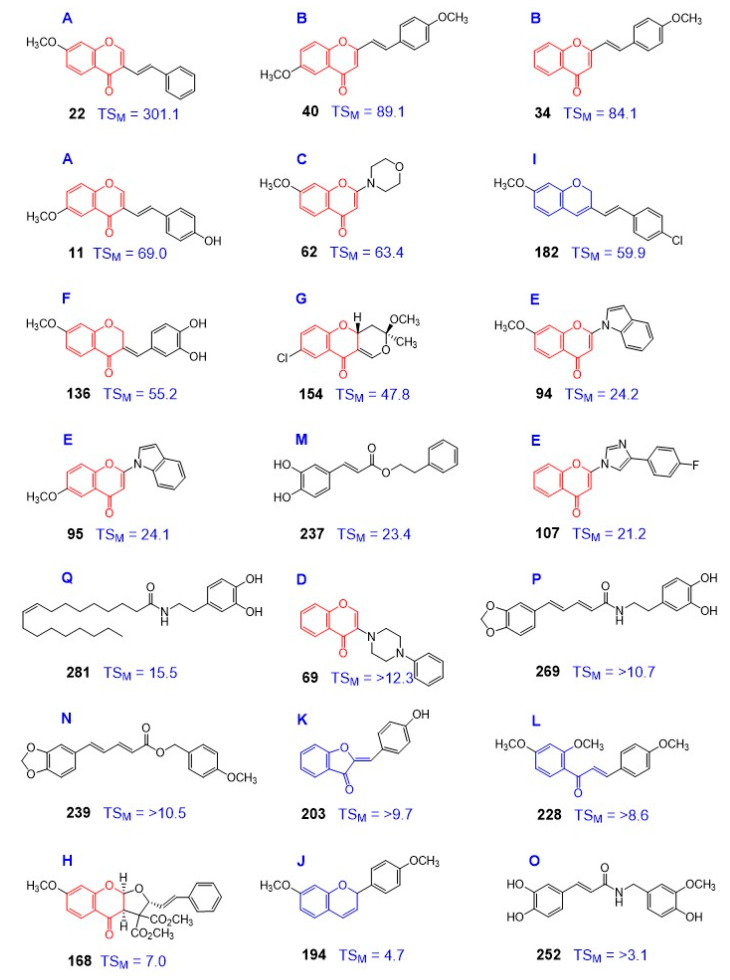
The most active compounds in each group, line-upped in the decreasing order of potency.

The most active compounds in each group are shown in Figure 7. Their cytotoxicity against human four OSCC cell lines, and three human normal oral mesenchymal (HGF, HPLF, and HPC), two human epithelial cells (HOK and HGEP), and tumor specificity (TSM (determined with OSCC vs. human normal mesenchymal cells), TSE) (determined with OSCC vs. human normal epithelial cells) are shown in Table 5.

Further, 7-methoxy-3-[(1*E*)-2-phenylethenyl]-4*H*-1-benzopyran-4-one (compound **22**) showed the highest TS value (TS_M_ = 301.1), followed by 2-[(1*E*)-2-(3,4-dimethoxy)ethenyl]-4*H*-1-benzopyran-4-one (compound **40**) (TS_M_ = 89.1) > 2-[(1*E*)-2-(4-methoxyphenyl)ethenyl]-4*H*-1-benzopyran-4-one (compound **34**) (TS_M_ = 84.1) > (*E*)-3-(4-Hydroxystyryl)-6-methoxy-4*H*-chromen-4-one (compound **11**) (TS_M_ = 69.0) > 7-methoxy-2-(4-morpholinyl)-4*H*-1-benzopyran-4-one (compound **62**) (TS_M_ = 63.4) > (*E*)-3-(4-cholorostyryl)-7-methoxy-2*H*-chromene (compound **182**) (TS_M_ = 59.9) > (3*E*)-2,3-dihydro-3-[(3,4-dihydroxyphenyl)methylene]-7-methoxy-4*H*-1-benzopyran-4-one (TS_M_ = 52.2) (compound **136**). It is noted that these compounds showed comparable TS values of doxorubicin (DXR) and much higher TS value than 5-FU. It was unexpected that DXR and 5-FU showed potent toxicity against human epithelial cells such as human oral keratinocyte (HOK) and human progenitor of human gingival epithelial cells (HGEP) (c/a and d/a in Table 5). We have reported previously that DXR induced apoptosis (characterized by the loss of cell surface microvilli, chromatin condensation, nuclear fragmentation, and caspase-3 activation) in these keratinocytes [54]. On the other hand, compounds **11**, **22**, **34**, **62,** and **69** showed much lower keratinocyte toxicity (Table 5).

### 3.4. Mechanism of Action

Compounds **11** and **22** in 3-styrylchromones (**A**), **34** and **40** in 2-styrylchromones (**B**), **95** in 2-azolylchromones (**E**), **228** in chalcones (**L**), and **237** in cinnamic acid phenethyl esters (**M**) induced apoptosis [caspase-3 activation (assessed by western blot analysis) and subG1 cell accumulation (assessed by cell sorter analysis)] in human OSCC cell lines. On the other hand, compounds **62** in 2-(*N*-cyclicamino)chromones (**C**), **95** in 3-(*N*-cyclicamino)chromones (**D**), **107** in 2-azolylchromones (**E**), **154** in pyrano[4,3-*b*]chromones (**G**), and **168** in furo[2,3-*b*]chromones (**H**) did not induce apoptosis (Table 6). Compounds **22**, **34**, **40,** and **107** also induced G2 + M cell accumulation, but only the first 3 compounds induced apoptosis. This indicated that the induction of G2 + M accumulation itself does not guarantee the induction of apoptosis.

### 3.5. Other Biological Actions of Chromones, Esters, and Amides

We searched other biological activities of chromones, esters, and amides (Supplementary Table S2). Table 7 listed up the most potent compounds that showed biological activity higher than positive controls. Compounds **10, 12**, **15**, **124, 136**, **229**, **231**, **237**, **258,** and **261** scavenged the DPPH radical, more potently than ascorbic acid, a well-known antioxidant [23], suggesting its antioxidant action.

Compounds **10**, **14**, **15**, **18**, **131**, **132**, **136**, **266,** and **267** inhibited α-glucosidase (EC 3.2.1.20) that is responsible in breaking down starch and disaccharides to glucose, more potently than acarbose. This suggest their possible antihyperglycemic effect.

Compounds **10**, **15**, **124**, and **136** show both α-glucosidase inhibitory and antioxidant actions, suggestion that they can be lead compounds for manufacturing as antidiabetic drugs.

Compounds **38**, **39**, **87**, **89**, **153**, **173**, **177,** and **236** inhibited monoamine oxidase (MAO-B) more effectively than pargyline, an irreversible selective MAO-B inhibitor drug. This suggests their application to treat the Parkinson’s disease and Alzheimer’s disease [24,27,28,30,55]. Halogen-containing compounds show more potent inhibitory activity. All compounds showed higher MAO-B-specific inhibition than positive controls and, therefore, were not likely to exert adverse effects due to MAO-A inhibition. Furthermore, they show reversible inhibition and thus were much convenient for the sudden interruption of treatment, as compared with irreversible inhibitors.

Compounds **230** and **235** inhibited the butyrylcholinesterase (BChE) more potently than neostigmine, suggesting that they may serve as lead compounds for the development of novel BChE inhibitors and candidate lead compounds for the prevention or treatment of Alzheimer’s disease [55].

We found that all compounds tested showed no anti-HIV activity (SI < 1), in contrast to popular anti-HIV substances (dextran sulfate, curdlan sulfate, azidothymidine, 2′,3′-dideoxycytidine, azidothymidine, and 2′,3′-dideoxycytidine) (SI = 53–2512) (Supplementary Table S3).

## 4. Serious Problems of Neurotoxicity in G2 + M Blocker

We found that highly tumor-specific 3-styrylchromone derivatives [7-methoxy-3-[(1*E*)-2-phenylethenyl]-4*H*-1-benzopyran-4-one (compound **22**) and 3-[(1*E*)-2-(4-hydroxyphenyl)ethenyl]-7-methoxy-4*H*-1-benzopyran-4-one (compound **29**)] (TS_M_ = 301 and 182, respectively) (Supplementary Table S1) induced subG1 and G2 + M arrest [35]. We also have recently reported that several G2/M blockers such as taxanes paclitaxel (Taxol^®^, the first microtubule stabilizing agent [57]) and docetaxel, show very high TS_M_ values (>7267 and >86,122, respectively) [58]. Marinho et al. reported recently that 4′-methoxy-2-styrylchromone induced mitotic arrest in human tumor (human Caucasian breast adenocarcinoma MCF-7 and human lung adenocarcinoma NCI-H460) cell lines, in a similar fashion to paclitaxel [59]. Soo et al. reported that cudraflavone C (Cud C), a naturally occurring flavonol, induced apoptosis (caspase activation) in colorectal cancer cells (CRC) and tumor-selective cytotoxicity by targeting the PI3K-AKT pathway [60].

However, many reports, including ours, demonstrated that microtubule-targeted agents have potent neurotoxicity, adversely affecting the quality of life of patients on a long-term basis [61,62,63,64]. Iijima et al. recently reported that carboplatin (CBDCA) was highly neurotoxic (TS_N_ = 0.11 (3.2/27.9)), calculated using the data of Table 2 in Ref. [64]. It is urgent to investigate the neurotoxicity, extravasation as well as stomatitis of chromone derivatives, esters, and amides.

## 5. Conclusions and Future Direction

We found that:

(i) Chromone showed much higher tumor specificity as compared with three major polyphenols.
(ii) A total 291 newly synthesized compounds of 17 groups (consisting of 12 chromones, 2 esters, and 3 amides) gave a wide range of the intensity of tumor specificity.
(iii) Their tumor specificity is correlated with chemical descriptors that reflect 3D structure and electric state.
(iv) 7-Methoxy-3-[(1*E*)-2-phenylethenyl]-4*H*-1-benzopyran-4-one (compound **22**), which belongs to 3-styrylchromones, showed the highest tumor specificity. Compound **22** induced subG1 and G2 + M cell population in human OSCC cell line, with much less keratinocyte toxicity as compared with doxorubicin and 5-FU. This compound can be used as a lead compound to manufacture more active compound.

It is crucial to identify the target molecules (Step 4 in Figure 1). To accomplish this, ^13^C-labeled compound **22** will be prepared, using 2-hydroxyacetophenone derivatives and ^13^C-dimethylformamide, or using ^13^C-iodomethane as methylation agent, and then the differential incorporation of ^13^C into malignant and nonmalignant cells will be investigated, with LC-MS. Compound **22**, labeled with fluorescence dye (Cy3, CY5, Cy7), will be tested to detect the intracellular uptake and distribution into organelles, using confocal laser microscopy. Binding of cellular protein to and elution from chromone-attached beads may be useful to identify the binding proteins.

In order to explore more potent chromone derivatives, the following three steps will be repeated: (i) prediction by QSAR of the best fit substituents that yield the highest TS_M_ and TSE, (ii) synthesis of compounds introduced with such predicted substituents, and (iii) confirmation of antitumor potential (Step 5). However, it is important to eliminate the compounds that show potent keratinocyte toxicity, neurotoxicity, and extravasation (Step 6), before animal experiment (Step 6) and clinical application (Step 7).

The present study demonstrated that only selected compounds that have the optimal 3D structure show the highest tumor specificity, whereas most of other analogs that have similar structure show much less tumor specificity (Supplementary Table S1). This suggests the presence of binding components or receptors for chromones. It remains to be investigated whether compounds **22** and **40** may interact with estrogen receptors, since these compounds have structural similarity with isoflavones (such as daidzein and genistein) and to some degree with tamoxifen, which have been used for the treatment of oral squamous cell carcinoma that express estrogen receptors [65,66,67]. In addition, it seems that the “para” like substitution is favorable, possibly because it mimics the structure of estrogen. It is highly probable that different groups of chromone-related compounds have different anticancer mechanisms depending on their structure (Table 6).

## Figures and Tables

**Table 1:** Incidence of oral mucositis and peripheral neuropathy induced by anticancer drugs.

		Reported Incidence (%)	
Classification	Drugs	Oral Mucositis	Peripheral Neuropathy
Vinca alkaloid	Vinorelbine (VNR)	15.2	12.2
	vinblastine (VBL)	0.4	2.2
	Vincristine (VCR)	0.1	25.5
Microtubule inhibitor	Eribulin (ERI)	39.5	24.7
Platinum	CDDP (cisplatin)	2.6	1.5
	L-OHP (oxaliplatin)	12.0	45.5
	CBDCA (carboplatin)	N.D.	N.D.
Taxane	DTX (docetaxel)	N.D.	N.D.
	PTX (paclitaxel)	18.2	55.0
	NabPTX (paclitaxel)	2.8	39.0
Antimetabolite	5-FU (fluorouracil)	6.7	0.2
	GEM (gemcitabine)	N.D.	N.D.
Topoisomerase inhibitor	IRT (irinotecan)	N.D.	N.D.
	ETP (etoposide)	N.D.	N.D.
Anthracycline	DOX (doxorubicin)	51.7	27.6
Molecular target drug	Rmab (ramucirumab)	54.3	N.D.
	Cmab (cetuximab)	>10.0	0.5~10.0
	Nmab (nivolumab)	1.0~5.0	3.1
Proteasome inhibitor	Bmib (bortezomib)	<5	28.3

N.D. no data reported.

**Table 2:** Classification of chemotherapeutic drugs according to tissue damage after extravasation.

Type of Damages	Principal Categories	Drugs
**Vesicants**	**DNA-binding compounds**	
	Anthracyclines	Amrubicin; Daunorubicin; Doxorubicin; Epirubicin;
		Idarubicin; Mitoxantrone; Pirarubicin
	Alkylating agents	Bendamustine; Busulfan; Carmustine; Melphalan;
		Nimustine; Ranimustine; Streptozocin
	Antitumor antibiotic	Actinomycin D
	Other	Trabectedin
	**Non-DNA-binding compounds**	
	Taxanes	Docetaxel; Paclitaxel; Nab-paclitaxel
	Vinka alkaloids	Vinblastine; Vincristine; Vindesine; Vinorelbine
	**Others**	
	Antibody–drug conjugate	Gemtuzumab ozogamicin
	Antitumor antibiotic	Mitomycin C
**Irritants**	Anthracyclines	Aclarubicin; Liposomal doxorubicin
	Alkylating agents	Dacarbazine; Cyclophosphamide; Ifosfamide; Temozolomide
	Antibody–drug conjugate	Trastuzumab emtansine
	Antimetabolites	Azacitidine; Gemcitabine; Fluorouracil; Tegafur
	Antitumor antibiotics	Bleomycin; Peplomycin
	Platinum salts	Carboplatin; Cisplatin Oxaliplatin; Nedaplatin; Miriplatin
	Proteasome inhibitor	Bortezomib
	Taxane	Cabazitaxel
	Topoisomerase I inhibitors	Irinotecan; Topotecan
	Topoisomerase II inhibitor	Etoposide
	Others	Arsenic Trioxide; Nelarabine; Picibanil; Porfimer; sodium
**Nonvesicants/** **Nonirritants**	Antimetabolites	Cladribine; Clofarabine; Cytarabine; Enocitabine
		Fludarabine; Methotrexate; Pemetrexed
	Antibody–drug conjugates	Brentuximab vedotin; Ibritumomab tiuxetan
	Antineoplastic agents	Degarelix; Fulvestrant; Goserelin; Leuprorelin
	Hormonal	
	Monoclonal antibodies	Alemtuzumab; Bevacizumab; Cetuximab; Ipilimumab;
		Mogamulizumab; Ofatumumab; Panitumumab; Pertuzumab; Ramucirumab; Rituximab; Trastuzumab
	Monoclonal antibodies (immune checkpoint inhibitors)	Atezolizumab; Avelumab; Durvalumab; Ipilimumab; Nivolumab; Pembrolizumab
	Others	BCG; Calcium folinate; Celmoleukin; Dexrazoxane; Eribulin; Interferon; L-asparaginase; Levofolinate;
		Octreotide; Pentostatin; Talaporfin sodium; Teceleukin

**Table 3:** Tumor specificity (TS) of polyphenols.

Compound	Number of Compounds	Mean TS^M^ (Range)
Lignin–carbohydrate complexes	4	2.7 (1.7~4.1) ^1^
Flavones, flavonols	36	1.2 (0.3~3.2)
Flavonoids	31	3.2 (0.8~31.7)
Isoprenyl flavonoids	22	2.1 (1.6~3.0)
Tricin, morin, quercetin, kaempferol	4	1.5(1~2.2)
Isoliquiritigenin, datiscetin, galangin	3	2.0 (1~4)
Resveratrol, daidzein, genistein	3	2.1 (1.1~2.9)
Gallic acid, catechin, epigallocatechin gallate	3	2.1 (1.0~4.1)
Procyanidins	6	4.8 (1.0~7.4)
Hydrolyzable tannins (monomer)	7	1.5 (1.0~2.5)
Hydrolyzable tannins (oligomers)	3	1.4 (1.2~1.5)
Large circular ellagitannins	4	4.4 (2.3~8.2)
2-Styrylchromones	6	7.3 (1.1~17.4)
3-Styrylchromones	15	14.9 (1.6~69.0)
Anthracyclines	4	181 (47~259)

^1^ Cited from [22].

**Table 4:** Molecular shape is the key determinant of tumor specificity.

	Chemical Descriptors That Correlate with Tumor Specificity	Ref.
3-Styrylchromones (**A**)	Molecular shape, electrostatic interaction, charge	[33,35]
2-Styrylchromones (**B**)	Molecular shape and flatness	[37]
2-(*N*-Cyclicamino)chromones (**C**)	Molecular shape, 3D-structure	[38]
3-(*N*-Cyclicamino)chromones (**D**)	3D-structure, lipophilicity	[39]
2-Azolylchromones (**E**)	3D/topological shape, size, polarizability, lipophilicity	[40,41]
3-Benzylidenechromones (**F**)	Molecular shape, size, polarization	[42]
Pyrano[4,3-*b*]chromones (**G**)	3D structure, polarity, ionic potential, electric state	[43]
Furo[2,3-*b*]chromones (**H**)	Molecular flexibility, density, size and shape, lipophilicity	[44]
3-Styrylchromenes (**I**)	Molecular shape and flatness	[45]
Aurones (**K**)	Molecular shape, size, polarizability	[46]
Chalcones (**L**)	Molecular shape and polarization	[47]
Cinnamic acid phenethyl esters (**M**)	Shape, size and ionization potential	[48]
Piperic acid esters (**N**)	Molecular shape, size, ionization potential, electronegativity	[49]
Phenylpronanoid amides (**O**)	Molecular size (surface area), electrostatic interaction	[50]
Piperic acid amides (**P**)	Molecular shape, electrostatic interaction	[51]
Oleoylamides (**Q**)	Molecular polarization and hydrophobicity	[52]

**Table 5:** Tumor specificity and keratinocyte toxicity of chromones and anticancer drugs.

		CC_50_ (μM)							
			Normal Oral Cells						
		Four	Mesen-	Epithelial					
		OSCCs	chymal	HOK	HGEP	TS_M_	TS_E_		
Group	Compd.	(a)	(b)	(c)	(d)	(b/a)	(c/a)	(d/a)	Ref.
3-Styrylchromones (**A**)	**11**	2.0	138	19.0	800	**69.0**	9.5	**400.0**	[33]
3-Styrylchromones (**A**)	**22**	0.6	182		400	**301.1**		**662.1**	[35]
2-Styrylchromones (**B**)	**34**	1.9	159		100	**84.1**		**52.8**	[37]
2-Styrylchromones (**B**)	**40**	3.8	336			**89.1**			
2-(*N*-Cyclicamino)chromones (**C**)	**62**	5.5	348	357.7		**63.4**	**65.2**		[38]
3-(*N*-Cyclicamino)chromones (**D**)	**69**	32.3	>397	400.0		>12.3	**12.4**		[39]
2-Azolylchromones (**E**)	**94**	6.3	153			24.2			[40]
2-Azolylchromones (**E**)	**95**	1.5	36			24.1			
2-Azolylchromones (**E**)	**107**	18.4	389			21.2			[41]
3-Benzylidenechromones (**F**)	**136**	7.3	>400	3.8	3.3	**55.2**	0.5	0.5	[42]
Pyrano[4,3-*b*]chromones (**G**)	**154**	5.0	240	20.3		47.8	4.1		[43]
Furo[2,3-*b*]chromones (**H**)	**168**	37.2	261			7.0			[44]
3-Styrylchromenes (**I**)	**182**	4.7	280			**59.9**			[45]
3-Flavens (**J**)	**194**	73.3	348			4.7			
Aurones (**K**)	**203**	41.4	>400			>9.7			[46]
Chalcones (**L**)	**228**	<4.4	38			>8.6			[47]
Cinnamic acid phenethyl esters (**M**)	**237**	8.5	199			23.4			[48]
Piperic acid esters (**N**)	**239**	37.9	>400			>10.5			[49]
Phenylpronanoid amides (**O**)	**252**	122.0	378			>3.1			[50]
Piperic acid amides (**P**)	**269**	75.0	>800			>10.7			[51]
Oleoylamides (**Q**)	**281**	0.6	9.7	2.5	0.4	15.5	4.0	0.6	[52]
DXR		0.1	9.7	0.1	0.027	**121.8**	**1.5**	**0.3**	[35,39]
5-FU		61.8	1000.0	24.7	18.8	16.2	**0.4**	**0.3**	[35]

**Table 6:** The most active compounds in each group do not necessarily induce apoptosis in human oral squamous cell carcinoma (OSCC) cell line.

Group	Compd.	Mechanism of Action	Ref.
3-Styrylchromones (**A**)	**11**	Mitochondrial vacuolization caspase-3 ↑	[34]
3-Styrylchromones (**A**)	**22**	subG1↑ G2 + M↑	[35]
2-Styrylchromones (**B**)	**34**, **40**	subG1↑ G2 + M↑	[37]
2-(*N*-Cyclicamino)chromones (**C**)	**62**	No apoptosis cytotoxic	[38]
3-(*N*-Cyclicamino)chromones (**D**)	**69**	No apoptosis cytostatic	[39]
2-Azolylchromones (**E**)	**95**	Caspase-3↑	
2-Azolylchromones (**E**)	**107**	G2 + M phase cells↑ No apoptosis cytostatic	[41]
Pyrano[4,3-*b*]chromones (**G**)	**154**	No apoptosis cytostatic	[43]
Furo[2,3-*b*]chromones (**H**)	**168**	No apoptosis	[44]
Chalcones (**L**)	**228**	Caspase-3↑	[47]
Cinnamic acid phenethyl esters (**M**)	**237**	Caspase-3↑	[48]

**Table 7:** Oher biological activities of chromones, esters, and amides.

			EC_50_ or IC_50_ (μM)		
Group	Inhibition of	Compd. No	Compd.	Positive Control	Ref.
3-Styrylchromones (**A**)	DPPH radical	**10**, **12**, **15**	17, 22, 23	23 (ascorbate)	[23]
	α-glucosidase	**10**, **14**, **15**, **18**	16, 9, 10. 16	>100 (acarbose)	[23]
2-Styrylchromones (**B**)	MAO-B	**38**, **39**	0.017, 0.024	0.22 (Pargyline)	[24]
2-Azolylchromones (**E**)	MAO-B	**87**, **89**	0.028, 0.019	0.22 (Pargyline)	[26]
3-Benzylidenechromones (**F**)	DPPH radical	**124**, **136**	13, 13	12 (ascorbate)	[27]
	α-glucosidase	**131**, **132**, **136**	15, 25, 28	900 (acarbose)	[27]
Pyrano[4,3-*b*]chromones (**G**)	MAO-B	**153**	0.2	0.22 (Pargyline)	[28]
3-Styrylchromenes (**I**)	MAO-B	**173**, **177**, **181**	0.010, 0.015, 0.016	0.22 (Pargyline)	[30]
Cinnamic acid phenethyl esters (**M**)	DPPH radical	**229**, **231**, **237**	18, 11, 18	23 (ascorbate)	[55]
	MAO-B	**236**	0.013,	0.22 (Pargyline)	[55]
	BChE	**230**, **235**	4.9, 6.8	7.1 (Neostigmine)	[55]
Phenylpronanoid amides (**O**)	DPPH radical	**258**, **261**	8.7, 8.1	12 (ascorbate)	[55]
	α-glucosidase	**266**, **267**	30, 29	900 (acarbose)	[56]

## Supplementary Materials

The following are available online at https://www.mdpi.com/2305-6320/7/9/50/s1, Table S1: Tumor specificity of 291 compounds; Table S2: Radical scavenging and monoamine oxidase inhibitory and cholinesterase inhibitory activities of chromones, esters, and amides; Table S3: Test for anti-HIV activity of chromones, esters, and amides.
